# The functional significance of dentin sialoprotein-phosphophoryn and dentin sialoprotein

**DOI:** 10.1038/s41368-018-0035-9

**Published:** 2018-11-05

**Authors:** Helena Ritchie

**Affiliations:** Department of Cariology, Restorative Sciences and Endodontics, University of Michigan School of Dentistry, Ann Arbor, USA

## Abstract

Phosphophoryn (PP) and dentin sialoprotein (DSP) are the most dominant non-collagenous proteins in dentin. PP is an extremely acidic protein that can function as a mineral nucleator for dentin mineralization. DSP was first identified in 1981, yet its functional significance is still controversial. Historically, these two proteins were considered to be independently synthesized and secreted by dental pulp cells into the developing dentin matrix. However, with the identification of the DSP coding sequence in 1994, followed 2 years later by the finding that the PP coding sequence was located immediately downstream from the DSP sequence, it became immediately clear that DSP and PP proteins were derived from a single DSP-PP (i.e., dentin sialophosphoprotein, DSPP) transcript. Since DSPP cDNA became available, tremendous progress has been made in studying DSP-PP mRNA distribution and DSP generation from the DSP-PP precursor protein at specific cleavage sites by protease tolloid-related-1 (TLR1) or bone morphogenetic protein 1 (BMP1). The functions of DSP-PP and DSP were investigated via DSP-PP knockout (KO) and DSP knockin in DSP-PP KO mice. In addition, a number of in vitro studies aimed to elucidate DSPP and DSP function in dental pulp cells.

## Endodontics: Functional significance of dentin sialoprotein-phosphophoryn and dentin sialoprotein

Along with phosphophoryn (PP), dental sialoprotein (DSP) is the dominant non-collagen protein in dentin, and in vitro studies have demonstrated that DSP is involved in inducing the differentiation of dental pulp cells into odontoblast-like cells, which form dentin. PP is known to be involved in the mineralization of dentin, but the functional significance of DSP had been controversial. Helena Ritchie of the University of Michigan School of Dentistry conducted a review of studies investigating the derivation, function and distribution of PP and DSP. It was originally thought that PP and DSP were synthesized independently; later, it became evident that they derive from a single DSP-PP gene. Wider DSP-PP distribution in various tissues, including kidney and salivary glands, and DSP or PP expression in non-mineralized tissues suggest that the proteins may have functions other than mineralization.

## Introduction

Prior to 1990, early efforts to understand dentin mineralization focused on analyzing the components of dentin using classical protein isolation and characterization techniques. The major component of mineralized tissues, such as bone and dentin, was found to be collagen (Col) type I. In addition to collagenous proteins, acidic non-collagenous proteins were identified and postulated to play significant roles during tissue mineralization. For example, dentin sialoprotein (DSP) and phosphophoryn (PP) were found to be the two most abundant acidic non-collagenous proteins in dentin.^1,2^ PP was identified in 1967 by Veis and Perry.^3^ PP is an extremely acidic protein and well established as a mineral nucleator for dentin mineralization.^4,5^ DSP was identified in 1981.^6^ As DSP shares similar composition to bone sialoprotein (BSP), it was named dentin sialoprotein. Osteopontin (OPN) and BAG-75 also share a similar composition to DSP. The N-terminal amino acid sequence of DSP was later found to be IPVPQLVP.^1,7^

## DSP cDNA cloning

Using a gt11 expression library and anti-DSP monoclonal antibodies, two DSP cDNAs were isolated and sequenced.^8^ The shorter DSP cDNA sequence contained 750 nucleotides coding for 244 amino acids, including a leader sequence and partial DSP coding sequence. The longer isolated DSP cDNA sequence contained 1 200 nucleotides that coded for 366 amino acids, including the leader sequence and a DSP coding sequence. The N-terminal amino-acid sequence (i.e., IPVPQLVPL) from DSP cDNA was identical to the reported N-terminal amino-acid DSP sequence determined by Edman degradation. The deduced amino-acid compositions from DSP cDNA were similar to those of the earlier isolated DSP glycoproteins (i.e., 350 amino acids), which were based on sedimentation equilibrium measurements. This long cDNA sequence was shown to code for rat DSP.^8^

## Availability of DSP cDNA enables identification of the PP coding sequence at the 3′ end of DSP and isolation of the DSP-PP gene

**Fig. 1:**
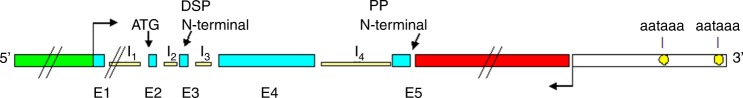
Rat DSP-PP Genomic Organization. The rat DSP-PP gene is distributed in five exons and four introns. E1, exon 1, the 5′ noncoding sequence; E2, exon 2, the 5′ noncoding region, the leader sequence and the N-terminal two amino acids for DSP; E3, exon 3, the DSP coding sequence; E4, exon 4, the DSP coding sequence; E5, exon 5, the C-terminal DSP coding sequence and the mature PP sequence (i.e., 1569 bp) as well as the 3′ noncoding sequence (i.e., 1523 bp); I_1_, intron 1; I_2_, intron 2; I_3_, intron 3; I_4_, intron 4. Two polyadenylation sites (aataaa) were represented by ;  Represents DSP exons;  Represents PP exon;  Represents the 3′ noncoding sequence;  Represents the DSP-PP promoter region;  Represents the transcription start site;  Represents the stop codon

**Fig. 2:**
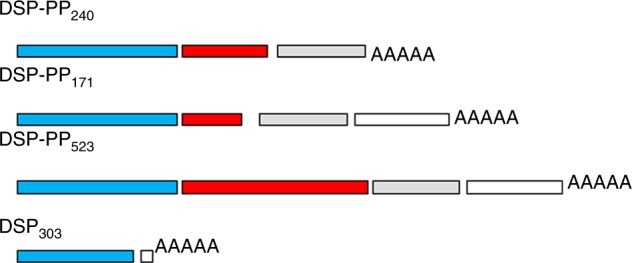
Multiple DSP-PP and DSP-only transcripts from the rat DSP-PP gene. The DSP-PP_240_ transcript, using the first polyadenylation site, will generate a mature DSP protein and a PP_240_ isoform. DSP-PP_523_ and DSP-PP_171_ transcripts, using the second polyadenlyation site, will give rise to a mature DSP protein, a mature PP_523_, and a mature DSP protein and PP_171_ isoform. The DSP-only transcript will produce a DSP_303_ protein

During analysis of the 3′ end of DSP cDNA by RT-PCR, Ritchie and Wang^9^ discovered an open reading frame with a size of 801 bp. This open reading frame was found to encode a putative leader sequence and a very acidic mature protein sequence with an amino-acid composition that coincided with the amino-acid composition of PPs from humans, cows, rats, and rabbits. Moreover, this deduced N-terminal sequence exactly matched those obtained from native rat PP by Linde et al.^2^ (4 amino acids) and by Chang et al.^10^ (14 amino acids), thus further supporting our claim that the cloned rat PP cDNA did indeed encode the expressed rat dentin PP protein. Most interestingly, this 801 bp PP sequence was later found to represent one of three DSP-PP multiple transcripts.^11,12^ We also showed DSP-PP arrangement at the genomic level.^13^ Rat DSP-PP cDNA was confirmed as a continuous open reading frame.^14^ MacDougall et al.^15^ described mouse dentin sialophosphoprotein (DSPP) cDNA. Also reported were DSP-PP (aka DSPP) cDNAs from humans, rats, and pigs.^11,12,16,17^ The rat DSP-PP gene is composed of five exons and four introns (Fig. 1).^12^ From the rat DSP-PP gene, three DSP-PP transcripts (i.e., DSP-PP_240_,^9,13^ DSP-PP_171_^11^, and DSP-PP_523_^12^) and the DSP only transcript^18^ were detected in day 5 tooth germ cDNAs (Fig. 2).

## Tissue distribution of the DSP protein and DSP-PP mRNA

Early immunohistochemical studies showed that DSP was localized to odontoblasts and pulp cells and was present in predentin and dentin but not in other tissues or cells, such as enamel, bone, muscle, or cartilage.^1,19,20^ In situ studies with DSP or DSP-PP riboprobes showed that DSP or DSP-PP mRNA was present in odontoblasts and preameloblasts.^21,22^ Early on, both DSP and PP proteins were considered to play a role in dentin mineralization because DSP or DSP-PP expression was associated with young odontoblasts and odontoblasts during dentin mineralized tissue formation.

The role of PP during dentin mineralization has been well established.^23^ PP is synthesized by odontoblasts and secreted at the mineralization front via odontoblastic processes, which suggests a unique role for PP in the conversion of uncalcified predentin to calcified dentin.^4,5^ DSP has been reported to be a weak inhibitor of dentin mineralization.^24^ However, the role of DSP is still not clear. Although DSP-PP expression was originally reported to occur predominantly in odontoblasts, later studies demonstrated wider DSP-PP expression in various tissues, including the periodontium,^25^ alveolar bone, basioccipital bone, skeletal bones, ribs, vertebrae, limb and kidney,^26^ salivary glands and hair follicles,^27^ lungs,^28^ and cartilage.^29^

For example, Baba et al.^25^ conducted immunohistochemical studies and demonstrated that DSP is localized not only in odontoblasts, dentin ECM, and preameloblasts, but also in alveolar bone, cellular cementum, osteocytes, cementocytes, and their matrices. Their in situ hybridization results also showed DSP expression in osteoblasts of alveolar bone, fibroblasts in the periodontal ligament, and cementoblasts in the cellular cementum.

## DSP and PP coding sequences are in one DSP-PP transcript, necessitating the investigation of (1) the location of DSP-PP cleavage site and (2) the protease responsible for this cleavage

Since the reports demonstrating that DSP and PP are derived from a DSP-PP gene were published, numerous efforts have focused on the DSP-PP cleavage site and on the protease responsible for DSP-PP cleavage. For example, in 2001, Qin et al.^30^ reported Y438 as the major cleavage site using tryptic fragments from native, purified DSP and proposed Phex as a protease for processing dentin matrix protein 1 (DMP1) and DSP-PP.^31,32^ In 2004, Steiglitz et al.^33^ reported that bone morphogenetic protein 1 (BMP1) was responsible for DMP1 cleavage but did not test DSP-PP cleavage because the DSP-PP precursor protein was not available in sufficient quantities for such studies. In 2010, Sun et al.^34^ reported that a D448A mutation blocked cleavage of recombinant mouse DSP-PP in a cultured human cell system and concluded that the key cleavage site was G447|D448. Also in 2010, von Marschall and Fisher^35^ used LoVo cells to secrete an intact mouse DSP-PP precursor that could be cleaved by adding BMP1. However, because of the low amounts of purported DSP-PP and DSP that were detected, no mass spectrometry or N-terminal sequence data were available to completely identify the precise cleavage site.

**Fig. 3:**
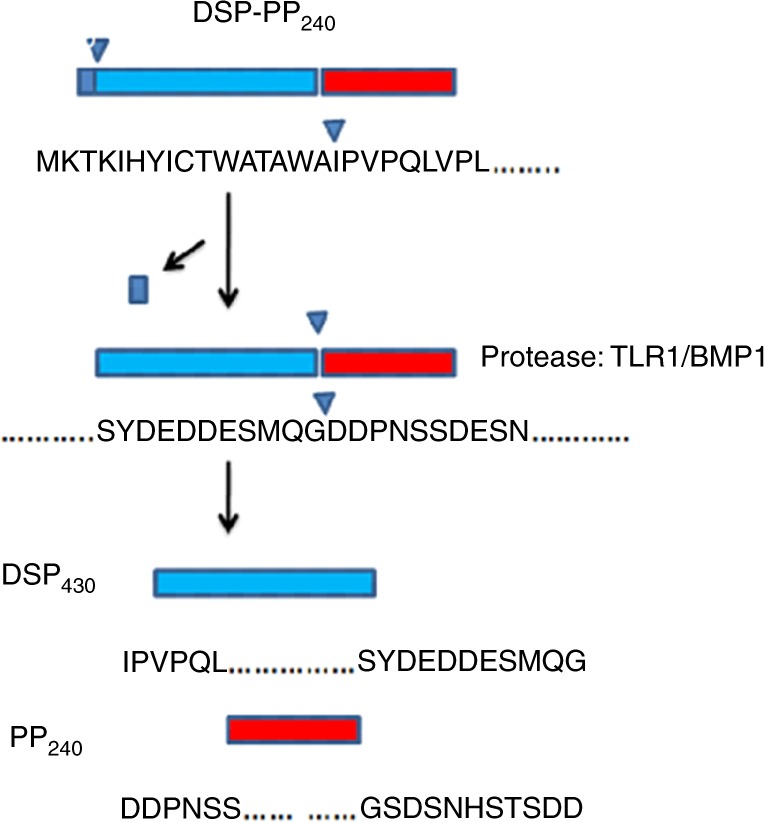
DSP-PP_240_ precursor protein is generated by translating the DSP-PP_240_ transcript. This precursor protein undergoes post-translational modifications, such as signal peptide cleavage, Asn-linked glycosylation, phosphorylation, and finally proteolytic processing (via tolloid-related-1 protein (TLR1) in insect cells/BMP1 in mammalian cells) to produce mature DSP_430_ and PP_240_

In view of the problem of obtaining sufficient DSP-PP protein from tissues and mammalian cells to sequence for these protease cleavage studies, insect Sf9 cells are routinely used to evaluate the expression of recombinant proteins encoded by baculovirus vectors. A baculovirus expression system was used to produce high yields of DSP-PP_240_ precursor protein that could be identified unambiguously by mass spectrometry.^36^ From MS/MS analysis of isolated tryptic fragments, a 76-amino-acid peptide was found to contain the PP N-terminal sequence of the PP_240_ band, and the DSP-PP cleavage site was proposed to be G447|D448.^36^ In 2012, using MS and MS/MS for analysis, a smaller, chymotryptic fragment (i.e., a 34-amino-acid peptide) was found, which contained the PP N-terminal sequence of the PP_240_ band. This permitted direct determination of the amino-acid sequence of this peptide by ion trap/fragmentation MS and firmly established the initial cleavage site in DSP-PP_240_ as G447|D448 (Fig. 3). Cleavage of DSP-PP_240_ at this site occurs after secretion into the conditioned medium of Sf9 cells, and cleavage was demonstrated to be catalysed by an endogenous Zn-dependent proteolytic activity secreted by Sf9 cells. Further analysis showed that the Sf9 cells transcribed a tolloid-related-1 (TLR1) peptidase gene (Spodoptera frugiperda tlr1) (Fig. 3). The human homologue of TLR-1, BMP1 can also cleave DSP-PP_240_ to release DSP_430_ and PP_240_^37^(Fig. 3). In 2013, the efficiency of DSP-PP processing was reported to be affected by mutations both flanking and distant from the cleavage site, suggesting that the residues near and distant from the cleavage site may have evolved to regulate DSP-PP processing.^38^

## Is DSPP cleavage critical for dentinogenesis?

To examine whether DSPP (i.e., DSP-PP) cleavage is required for dentin mineralization to proceed, Qin’s group showed that the normal DSPP transgene fully rescued dentin defects in *Dspp-*KO mice. (Note: DSPP null mice details are listed below.) However, a mutated D452A-DSPP transgene (i.e., where DSPP cleavage was mostly blocked) was not able to rescue the dentin defect. Thus, proteolytic processing of DSPP is a necessary activation step essential to its biological function in dentinogenesis.^39^

## Mutations in the DSP-PP (i.e., DSPP) gene are linked to dentinogenesis imperfecta I and II

Dentinogenesis imperfecta I and II (DGI I and II) have been linked to mutations of the DSPP gene. For example, Xiao et al. identified three mutations in the DSPP gene in dentinogenesis imperfecta patients.^40^ Two missense mutations were located in exons 2 and 3 of DSPP with progressive sensorineural high-frequency hearing loss (gene *DFNA39*). These missense mutations likely affected the cleavage of the DSPP signal peptide and thus damaged the generation of DSP and PP. A transition mutation at the donor-splicing site of intron 3 was found in one family without *DFNA39*, a mutation predicted to result in the skipping of exon 3. This skip mutation likely generated defective DSP protein and resulted in defective dentin. Furthermore, Zhang et al. reported a nonsense mutation (Gln45stop) in exon 3 of the *DSPP* gene in a Chinese family with dentinogenesis imperfecta Shields type II (DGI-II).^41^ This nonsense mutation likely generated a short DSP protein without PP expression. Thus, the lack of the PP protein and the presence of a very short DSP protein likely resulted in discoloration and an obliterated pulp chamber.

## Use of DSP-PP knockout (KO) mice to examine DSP-PP function

To investigate the function of DSP-PP during dentin formation, DSP-PP knockout (KO) mice were generated by Kulkarni’s group.^42^ DSP-PP KO mice showed hypomineralized teeth, thin dentin, lower mineral density, and a large dental pulp chamber, similar to those from patients with dentinogenesis imperfecta III. In addition, these animals showed increased levels of biglycan and decorin, which are small leucine-rich proteoglycans, in the widened predentin zone and void spaces among the calcospherites in the dentin of DSP-PP knockout teeth. The authors postulated that DSP-PP or the cleavage products DSP or PP might therefore potentially regulate proteoglycan levels in dentin.

## What is the function of the DSP protein in vivo using DSP-PP KO mice containing a DSP transgene?

To further examine the role of DSP during dentinogenesis, Kulkarni’s group used 5.7-kb DSP-PP elements to drive DSP expression in the dentin phosphophoryn (DPP) (i.e., PP) cKO mice, in which only DSP is expressed in a DSP-PP KO background, resulting in conditional dentin phosphophoryn knockout (DPPcKO) mice. DSP-only expression (in DPPcKO teeth) with an eight-fold increase in DSPP mRNA compared to that of the wild type (wt) showed a partial rescue of the DSP-PP null phenotype, yielding a restored predentin width, absent irregular unmineralized areas in dentin, and decreased pulp exposure. Micro-computed tomography (micro-CT) analysis of DPPcKO molars further confirmed this partial rescue with significant recovery in the dentin volume but not in the dentin mineral density. The authors claimed that these results indicated distinct roles of DSP and DPP in dentin mineralization, with DSP regulating the initiation of dentin mineralization and DPP being involved in the maturation of mineralized dentin.^43^ It is an interesting and challenging idea worth further investigation. An alternative explanation for the role of DSP could be that the presence of DSP might regulate biglycan and decorin expression, which leads to a narrow predentin layer.

Another study on the role of DSP during dentinogenesis was performed by Gibson and co-workers using the Col type I promoter to drive DSP protein expression in DSP-PP null mice. Gibson et al.^44^ reported that Col type I promoter-driven DSP expression led to thinner dentin formation. Dentin was more poorly mineralized and remarkably disorganized in DSP-PP null mice than in the DSP-PP KO mice. The authors attributed this result to the NH_2_-terminal fragment of DSPP (i.e., DSP protein), which may inhibit dentin mineralization or may serve as an antagonist of the accelerating action of PP, thus preventing predentin from being mineralized too rapidly during dentinogenesis. This discrepancy between Kulkarni’s group and Gibson et al. regarding the role of DSP during dentin formation is intriguing.

Weinstock and LeBlond^45,46^ used ^33^P-phosphate to label phosphoprotein and ^3^H-proline to label collagen type I. They observed that the phosphoprotein took ~ 4 h to reach the mineralization front once secreted from odontoblasts. In contrast, collagen type I took ~ 24–30 h to reach the mineralization front. Collagen type I takes time to mature and form collagen fibres. Bleicher et al.^47^ showed that collagen type I and DSPP have different temporal developmental expression patterns.

One possible explanation for the discrepancy regarding the role of DSP during dentin formation could be that the Col type I promoter (used by Gibson’s group) and the DSPP promoter (used by Kulkarni’s group) have different effects on temporal DSP expression. For example, Col type I promoter-driven DSPP expression (i.e., 13-fold increased mDSPP expression compared to that in wt) in Gibson’s group was concurrent with Col type I expression in transgenic mice, which might have interfered with Col type I maturation. Thus, poor maturation of Col type I might have resulted in the thinner dentin formation observed in Gibson’s group. DSPP promoter-driven DSPP expression (i.e., eight-fold increased mDSPP expression compared to that in wt) followed the correct Col type I and DSPP temporal expression patterns. Therefore, Col type I maturation is still able to proceed normally. It would be interesting to compare collagen assembly and maturation between the DSP-PP KO/DSPP promoter-driven DSP transgene and the DSPP KO/Col type I promoter-driven DSP transgene.

To determine whether DSP-PP expression is related to DSP-PP function in vivo in tissues other than dentin, Gibson et al. examined whether the absence of DSP-PP protein affects periodontium formation. They reported that the loss of DSPP protein in DSP-PP null mice led to periodontal disease in mice.^48^ To further probe whether the DSP transgene in DSPP null mice could restore defective periodontium, Gibson et al. reported that overexpressing the NH_2_-terminal fragment (i.e., DSP protein) of DSP-PP aggravated periodontal defects in DSP-PP knockout mice.^49^ Thus, these authors concluded that DSP was not able to restore defective periodontium.

## The observation of developmental abnormalities (i.e., circular dentin formation within the dental pulp) and chondrocyte-like cells expressing Col type II and Sox9 in DSP-PP KO mice suggests that DSPP has a role in dental pulp cell differentiation

**Fig. 4:**
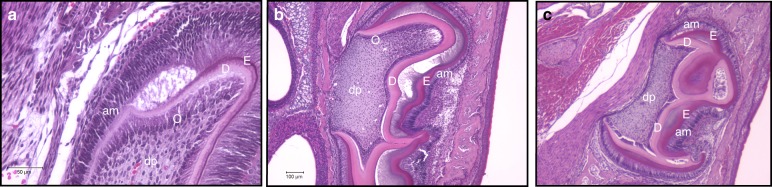
Altered epithelial/mesenchymal interactions in 1- and 6-day-old DSP-PP KO mice. The sections were stained with H&E. **a** 1-day-old DSP-PP KO M1 (400× magnification). **b** 6-day-old DSP-PP KO M1 (100× magnification). **c** 6-d DSP-PP KO M2 (100× magnification). Note: dentin formed a circular structure within the dental pulp in 6-day-old DSP-PP KO and the enamel and dentin layers were abnormally separated. M1, molar 1; M2, molar 2; am, ameloblast; dp, dental pulp; d, dentin; E, enamel; O, odontoblast

**Fig. 5:**
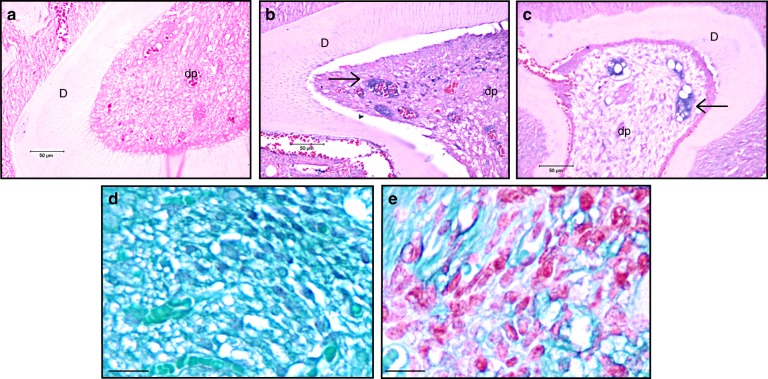
The presence of the chondrocyte marker collagen type II and acidic proteoglycan in 21-day-old DSP-PP KO mice. **a**–**c** Immunohistochemical (IHC) staining of 21-day-old wt M2 and 21-day-old DSP-PP KO M2/M3 sections using anticollagen type II antibodies at 400x magnification. **a** 21-day-old wt M2 section showed no antibody staining. **b** 21-day-old DSP-PP KO M2 section showed antibody staining in blue as indicated by the arrows. **c** 21-day-old DSP-PP KO M3 section showed antibody staining in blue as indicated by the arrows. **d**, **e** Sections were stained with Safranin O/fast green for the acidic proteoglycan at 1600x magnification. **d** 21-day-old wt M2 section showed no Safranin O staining in the dental pulp. **e** 21-day-old DSP-PP KO M2 showed Safranin O staining in the dental pulp

In 2014, Guo et al.^50^ reported that DSP-PP null mice had thinner dentin, a larger pulp chamber, lower mineral dentin density, and wider predentin than wt mice. Additionally, developmental abnormalities not previously reported were found in these DSP-PP null mice, such as circular dentin formation within dental pulp, altered epithelial/mesenchymal interactions (Fig. 4), and altered odontoblast differentiation, even as early as 1 day after birth.^50^ Surprisingly, chondrocyte-like cells were identified in the dental pulp from the teeth of KO mice. These chondrocyte-like cells expressed Col type II (Fig. 5b, c) and Sox9. No Col type II expression was detected in wt teeth (Fig. 5a). Safranin O staining demonstrated the presence of acidic proteoglycan (i.e., a chondrocyte marker) in the dental pulp obtained from 21-day-old DSP-PP KO M2 teeth (Fig. 5e). These developmental abnormalities found in DSP-PP KO mice suggest that expression of the DSP-PP protein or the cleavage product DSP or PP is required for normal odontoblast and ameloblast development. The absence of DSP-PP, DSP, or PP could not maintain the odontoblast lineage in DSP-PP KO mice.^50^

## DSPP function in vitro

Just as DSP-PP has been shown to affect cell differentiation in vivo, a number of in vitro studies have demonstrated similar effects on cell differentiation. For example, adipose-derived stromal cells (ADSC) are pluripotent cells. DSP-PP-expressing adenovirus (Ad-DSP-PP) was used to test the effects of DSP-PP on ADSC differentiation. The overexpression of DSP-PP promoted mineralization of ADSCs; together with the expression of early odontogenic marker genes, this finding implied that these cells may differentiate into functional odontoblast-like cells. These results suggested that DSP-PP or cleavage products DSP or PP could direct ADSC differentiation.^51^ This finding may be important for understanding tooth regeneration or reparative dentin formation.

## Recombinant human DSP protein induces human dental pulp cell differentiation into odontoblast-like cells

Lee et al.^52^ examined the effects of recombinant human dentin sialoprotein (rh-DSP; an ~ 90-amino-acid peptide located at the C-terminal region of DSP) in dental pulp cells. They added 10 ng•mL^−1^ of rh-DSP to human dental pulp cells and found enhanced cell proliferation, migration, increased alkaline phosphatase (ALP) activity, calcium nodule formation, and mRNA expression of odontoblastic markers, such as ALP, OPN, osteocalcin, DSPP, and DMP1. They reported that rh-DSP increased BMP-2 expression and Smad1/5/8 phosphorylation. The BMP2 antagonist noggin can block Smad 1/5/8 phosphorylation. rh-DSP phosphorylated extracellular signal-regulated kinase (ERK), c-Jun N-terminal kinase (JNK), Akt, and IκB-α, and induced nuclear translocation of the NF-κB p65 subunit. In summary, rh-DSP induces growth, migration, and differentiation in human dental pulp cells via the BMP/Smad, JNK, ERK, MAPK, and NF-κB signalling pathways.

## DSP protein binds to the integrin 6 receptor

To understand how the DSP protein can induce dental pulp cell differentiation into odontoblast-like cells, Wan et al.^53^ used DSP to screen a protein library and demonstrated that DSP binds to integrin 6. They further demonstrated that peptide DSP^aa183-219^ binds to integrin 6. This peptide promoted cell attachment, migration, differentiation, and mineralization of dental mesenchymal cells. In addition, DSP^aa183-219^ stimulated phosphorylation of ERK1/2 and P38 kinases. This activation was inhibited by an anti-integrin β6 antibody and siRNA. Furthermore, the authors showed that this DSP fragment induces SMAD1/5/8 phosphorylation and nuclear translocation via ERK1/2 and P38 signalling. SMAD1/5/8 binds to SMAD binding elements (SBEs) in the DSPP gene promoter. SBE mutations result in a decrease in DSPP transcriptional activity. Endogenous DSPP expression was upregulated by DSP^aa183-219^ (200 ng•mL^−1^) in dental mesenchymal cells.

## Summary

This review encompasses five topics: (1) DSP-PP distribution in various tissues, (2) DSP-PP precursor protein cleavage, (3) DSP-PP KO mouse models, (4) the effect of the DSP protein on DSP-PP KO mice, and (5) the effect of DSP-PP on adipose-derived stromal cells and the effect of DSP on dental pulp cells.

The presence of DSPP in odontoblasts suggests that DSPP functions in dentin mineralization. A wider DSP-PP expression has been found in various tissues, including the periodontium,^25^ alveolar bone, kidneys,^26^ and salivary glands, and DSP and PP expression has been found in non-mineralized tissues. Might there be other functions that DSP or PP have in these tissues beyond mineralization that have yet to be discovered?

DSP and PP proteins were generated from the DSP-PP precursor protein at specific cleavage sites by protease TLR1 or BMP1. The proteolytic processing of DSP-PP precursor is essential to dentinogenesis.^39^

DSPP null mice^42^ showed thinner dentin, a larger pulp chamber, lower mineral density, and wider predentin with increased biglycan and decorin expression. These results clearly demonstrate that the absence of the DSP-PP precursor protein and its cleavage products (i.e., DSP and PP) affect dentin mineralization. PP is well established as a nucleator for mineralization. The absence of PP is likely the cause of decreased mineral density. In addition, Guo et al. reported that DSP-PP KO mice did not maintain the odontoblast lineage and chondrocyte-like cells present in dental pulp that expressed Col type II and Sox9.^50^

To examine the function of the DSP protein in DSP-PP KO mice, the DSP transgene was introduced to DSP-PP KO mice with a DSP-PP promoter (by Kulkarni's group) and with a collagen type I promoter (by Gibson’s group). Interesting and controversial results were reported on the effects of DSP on DSP-PP KO mice (see the review under the section “What is the function of the DSP protein in vivo using DSP-PP KO mice containing a DSP transgene?”)

Guo et al. reported that DSPP KO mice did not maintain the odontoblast lineage.^50^ The inability to maintain the odontoblast lineage could lend support to the enlarged dental pulp chamber. Kulkarni’s group used the DSP transgene to partially rescue DSPP null mice, including restoring predentin width and recovering the dental volume. These findings could be due to the presence of the DSP protein, which might maintain the odontoblast cell lineage programme, so that Col I and other extracellular matrix proteins, such as Dmp1, osteocalcin, and OPN, are continually secreted. Future research is needed to verify whether the DSP protein could affect odontoblast lineage in vivo.

Adenovirus-driven DSPP protein overexpression was found to promote adipose-derived stromal cells to differentiate into functional odontoblast-like cells.^51^ The cleaved products DSP and PP likely affect the differentiation of these pluripotent cells. Lee et al.^52^ used the DSP (rh-DSP) protein to promote human dental pulp cell differentiation into odontoblast-like cells. The differentiation was via the phosphorylation of the ERK1/2 and P38 pathway. Wan et al.^53^ identified that DSP interacts with integrin 6 to induce SMAD1/5/8 phosphorylation and nuclear translocation via ERK1/2 and P38 signalling. Wan et al.^53^ demonstrated that the DSP domain ^aa183-219^ was responsible for activating these pathways, resulting in pulp cell differentiation into odontoblast cells. However, as shown by Lee et al.,^52^ rh-DSP (10 ng•mL^−1^) seems to be a more potent molecule for promoting pulp cell differentiation than the DSP^aa183-219^ peptide (200 ng•mL^−1^) described by Wan et al.^53^ The difference in molecular potency between the rh-DSP protein and the DSP peptide^aa183–219^ could be because these two DSP peptides were derived from different peptide locations.

Taken together, in vitro studies have demonstrated that DSP does have a role in inducing dental pulp cell differentiation in odontoblast-like cells.
